# A review of conspecific attraction for habitat selection across taxa

**DOI:** 10.1002/ece3.6922

**Published:** 2020-11-10

**Authors:** Valerie L. Buxton, Janice K. Enos, Jinelle H. Sperry, Michael P. Ward

**Affiliations:** ^1^ Department of Natural Resources and Environmental Science University of Illinois at Urbana‐Champaign Urbana IL USA; ^2^ Department of Evolution, Ecology, and Behavior University of Illinois at Urbana‐Champaign Urbana IL USA; ^3^ Engineer Research and Development Center Champaign IL USA; ^4^ Illinois Natural History Survey University of Illinois at Urbana‐Champaign Champaign IL USA

**Keywords:** conspecific attraction, conspecific cue, habitat selection, social information

## Abstract

Many species across taxa select habitat based on conspecific presence, known as conspecific attraction. Studies that document conspecific attraction typically provide social information (i.e., cues that indicate the presence of a given species) and then determine if a given species is more likely to settle at locations where the social information is provided compared to those locations that do not. Although the number of studies examining conspecific attraction has grown in recent years, a comprehensive review has not yet been undertaken. Here, we conducted a review of the literature and found 151 studies investigating conspecific attraction across eight taxa. We found that conspecific attraction is widespread with between 80% and 100% of studies, depending on taxa, documenting positive associations between habitat selection and the presence of conspecific cues. Conspecific attraction has been documented more frequently in bird and fish species with less attention given to invertebrate and mammal species. We use the patterns we found to (a) provide an overview of the current state of research on conspecific attraction and (b) discuss how important factors, such as cue characteristics and life history traits, may play a role in shaping conspecific attraction patterns within and across taxa.

## Graphical abstract

Many species across taxa select habitat based on conspecific presence, known as conspecific attraction. Although the number of studies examining conspecific attraction has grown in recent years, a comprehensive review has not yet been undertaken. Here, we conducted a review of the literature and found that conspecific attraction is widespread with between 80% and 100% of studies, depending on taxa, documenting positive associations between habitat selection and the presence of conspecific cues.

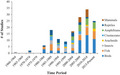

## 1 INTRODUCTION

Habitat selection by animals is not random, with evidence suggesting that animals use information to select locations with resources necessary for survival and reproduction (Schmidt et al., 2010). This information may be gathered either from physical cues, termed “non‐social information,” or through interactions with or observations of others in the environment, termed “social information” (Seppänen et al., 2007; Wagner & Danchin, 2010). Social information, either unintentionally or intentionally conveyed to others, can be gathered from many cues, including the physical presence of an individual, chemical cues, and/or acoustic vocalizations (Danchin et al., 2004; Wagner & Danchin, 2010). Indeed, many species across multiple taxa locate conspecifics with social cues and preferentially settle in these locations, often resulting in the phenomenon of conspecific attraction (reviews in Reed & Dobson, 1993; Stamps, 2001). However, the prevalence of conspecific attraction in relation to habitat selection and the differences in proximate mechanisms used to detect conspecifics across taxa have not yet been explored in a single review.

Using the presence of conspecifics to select habitat is considered widespread across taxa (e.g., Danchin et al., 2004) and is often studied under an adaptive framework considering the fitness‐related costs and benefits associated with the habitat selection strategy (e.g., Fletcher, 2006; Stamps, 2001). These benefits can include increased access to mates (Westneat & Sherman, 1997; reviewed in Kokko and Rankin 2006), increased protection from and defense against predators (Olsen et al., 2015; Roberts, 1996; but see Beauchamp, 2008), more efficient thermoregulation (Gilbert et al., 2010), and enhanced foraging efficiency (Sridhar et al., 2009; Stensland et al., 2003; Ward & Zahavi, 1973). Currently, the patchwork of individual, single‐species studies makes it difficult to discern how prevalent across taxa conspecific attraction actually is for habitat selection and what selective pressures are shaping the behavior within and across taxa.

The topic of conspecific attraction has been frequently reviewed within a taxa (e.g., birds: Ahlering & Faaborg, 2006; Ahlering et al., 2010; Szymkowiak, 2013; amphibians: Buxton & Sperry, 2017) or within special interests such as across species of conservation concern (Putman & Blumstein, 2019). On a similar note, heterospecific attraction, or using the presence of other species to select habitat, has been reviewed extensively across taxa (Mönkkönen et al., 1999; Putman & Blumstein, 2019; Seppänen et al., 2007). To our knowledge, however, there is no single literature review on conspecific attraction that synthesizes studies from multiple taxa to explore patterns of the habitat selection strategy across taxa. Such a literature review would greatly improve our understanding of why conspecific attraction occurs, and also identify knowledge gaps that future research should address.

Here, we conducted a literature review to explore and discuss patterns of conspecific attraction for habitat selection across several taxa. We use the resulting patterns generated to qualitatively investigate the following three questions:

1. Which broad taxonomic groups does conspecific attraction for habitat selection occur most frequently in?
2. How do the proximate mechanisms (e.g., chemical, acoustic, or visual cue) of conspecific attraction vary within and among taxa?
3. What are the fitness benefits gained by individuals using conspecific attraction (e.g., increased number of offspring), both within and across taxa?

We then discuss our results in light of how life history traits (such as mobility, dispersal, and sex‐ or age‐specific characteristics) could shape the proximate mechanisms of conspecific attraction. We also discuss how selection for or against the behavior could occur due to variation in life history traits both within and across taxa. Finally, within these discussion topics, we highlight research needs that would help fill in the gaps discovered in this review. This approach will therefore aid researchers in where to focus study efforts to better our understanding of conspecific attraction as a habitat selection strategy.

## 2 METHODS

We used Google Scholar and Web of Science to identify studies on conspecific attraction from 1960 to 2017 by specifying the keywords “conspecific attraction,” “conspecific information,” “conspecific cue,” and “social information.” From these publications, we also identified other relevant studies from their references. We limited our review to studies that identified conspecific attraction for purposes of breeding and/or nonbreeding habitat selection or the act of finding an area that is suitable to meet all of an organism's resource needs (i.e., the organism's “habitat”; Piper, 2011). We excluded studies that examined conspecific attraction to pursue specific intraspecific interactions, such as mate selection (e.g., Michelena et al., 2005; Pearl et al., 2000) and intrasexual territory defense (e.g., Campos et al., 2017), or to improve performance during a specific task, such as foraging decisions (e.g., van Bergen et al., 2004). We only reviewed experimental studies that manipulated conspecific presence with cue treatments to a habitat; these studies provide more direct evidence of conspecific attraction than observational studies (e.g., observing clustered distributions in a species; reviewed in Campomizzi et al. 2008).

We did not conduct a formal meta‐analysis, but instead used tallies from each publication to generate percentages of studies that found evidence of conspecific attraction. To address Question 1, we investigated eight taxonomic groups, with species grouped together by class: birds (Aves), ray‐finned fishes (Actinopterygii), reptiles (Reptilia), amphibians (Amphibia), mammals (Mammalia), insects (Insecta), arachnids (Arachnida), and crabs and lobsters (Malacostraca, hereafter referred to as “crustaceans”). We chose these groups because they had at least five published studies on conspecific attraction meeting the criteria described above. To address Question 2, within each study we categorized the cue(s) used to attract conspecifics that were experimentally manipulated. These cues included: acoustic cue (e.g., song or call playback), chemical cue, visual cue (e.g., decoys or models), conspecific presence (e.g., tethered or caged individuals), and indirect cues of conspecific presence (e.g., conspecific web and burrow). The conspecific presence cue included those studies in which the physical presence of conspecifics was experimentally used as a stimulus, and thus, one specific cue type was impossible to isolate. Some studies tested multiple cue types independently. For these studies, we considered each cue presented individually as a single “test.” We also categorized studies that used a combination of cues (e.g., acoustic playback and visual decoys combined).

To address Question 3, for those papers that found conspecific attraction, we categorized the proposed ultimate mechanisms (i.e., fitness benefits) of using conspecific cues for habitat selection based on discussion text in each study. We found that ultimate mechanisms could be categorized into the following six benefits to habitat selection via conspecific attraction: (1) location/identification of suitable habitat, (2) protection benefits (e.g., group defense from predators), (3) mating benefits (e.g., increased access to mates), (4) foraging benefits (e.g., enhanced foraging efficiency and sharing information about foraging locations), (5) thermoregulatory benefits, and (6) kin selection. To assess fitness benefits of conspecific attraction further and more directly, we identified all studies that quantified measures of fitness (e.g., survival, number of offspring produced, and clutch success/failure).

We acknowledge that we have not identified all studies fitting our criteria in this review, as the literature on conspecific attraction is vast. Moreover, a potential shortcoming of this review is that there is likely a positive‐results bias in publication, such that negative results from experimental tests are less likely to be published. We return to how this publication bias may affect our findings in the Discussion. Nonetheless, our search likely identified the majority of studies in the taxa we considered and provides an unbiased overview of the current state of published conspecific attraction research for the taxa of interest.

## 3 RESULTS

### 3.1 Question 1: How prevalent is conspecific attraction across taxa?

**FIGURE 1:**
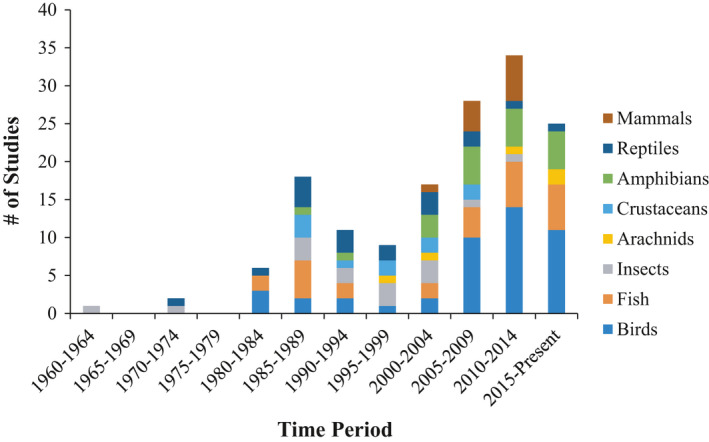
Timeline of conspecific attraction studies included in this review by taxonomic group from 1960 to 2017

**FIGURE 2:**
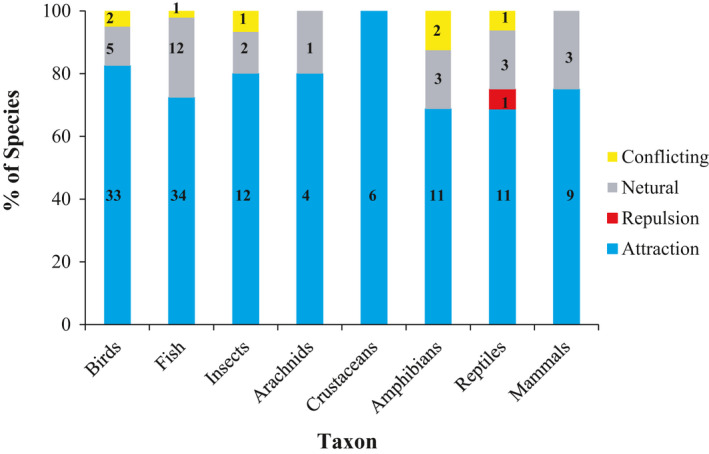
Percentage of species showing attraction, repulsion, no response (neutral), or conflicting response to conspecific cues grouped by taxa. Conflicting response indicates that a species showed a differential response to conspecific cues in two or more studies (e.g., one study showed attraction, while another showed repulsion). Species sample size for each category is listed in bold on the respective bar segment

We found a collective total of 151 publications on conspecific attraction for habitat selection (Figure 1, Appendix S1). Of the 157 species examined across studies, fish (30%) and birds (25%) comprised the greatest number of species examined, followed by amphibians (10%), reptiles (10%), insects (10%), mammals (8%), crustaceans (4%), and arachnids (3%). Most taxa showed a pattern of exhibiting conspecific attraction, including ≥ 80% of bird, insect, arachnid, and crustacean species and ≥ 65% of amphibian, reptile, mammal, and fish species (Figure 2). The majority of studies tested a single species (83%, 126/151).

### 3.2 Question 2: How do proximate mechanisms of conspecific attraction vary across taxa?

In birds, mammals, and amphibians, most tests involved the use of acoustic cues (birds: 79% of tests; mammals: 57%; amphibians: 42%). In reptiles, crustaceans, and fish, the dominant cue type tested was chemical cues (reptiles: 81% of tests; crustaceans: 53%; fish: 48%). In insects, the dominant cue type tested was visual cues (45% of tests), while in arachnids, the dominant cue type was indirect cues (e.g., presence of webs or silks; 50%). Across all cue types, chemical cues and presence cues were tested in 100% of taxa, acoustic and visual cues in 50% of taxa, and indirect cues in 38% of taxa. Only 12 studies used a combination of cue types in their experimental paradigm, and only in birds (20% of tests), amphibians (8% of tests), and insects (5% of tests).

**FIGURE 3:**
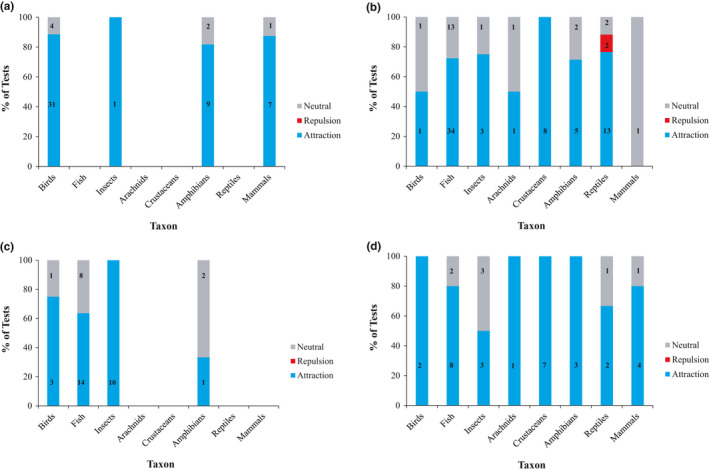
Percentage of tests in which taxa have shown conspecific attraction, repulsion, or no response (neutral) to (a) acoustic, (b) chemical, (c) visual, and (d) presence/abundance cues. If a study tested species response by multiple age classes or sexes to a given cue type or species response to multiple variations of a cue type and found attraction in any of these instances, then it was counted as a single test showing attraction. Sample size for each category is listed in bold on the respective bar segment. The absence of a bar for a taxon represents that there were no tests of that cue type

When considering acoustic cues only (i.e., not presented in combination with other cues), most studies on birds, mammals, and amphibians exhibited conspecific attraction in habitat treated with these cues (birds: 89% of tests; mammals: 88%; amphibians: 82%; Figure 3a). For chemical cues only, most studies on crustaceans, reptiles, insects, fish, and amphibians demonstrated conspecific attraction for habitat selection (crustaceans: 100% of tests; reptiles: 76%; insects: 75%; fish: 72%; amphibians: 71%). Fewer studies demonstrated conspecific attraction with chemical cues for arachnids (50% of tests; Figure 3b). For visual cues only, most studies on insects and birds found evidence for conspecific attraction with this cue type (insects: 100% of tests; birds: 75%; Figure 3c). Fewer studies found evidence of conspecific attraction using visual cues for fish (64% of tests) and amphibians (33% of tests; Figure 3c). For presence cues only, all studies on birds, amphibians, arachnids, and crustaceans (100% each of tests) found evidence of conspecific attraction with this cue type (Figure 3d). Most studies on fish and mammals (80% of tests) demonstrated conspecific attraction with presence cues, whereas fewer studies on reptiles (67% of tests) and insects (50% of tests) demonstrated conspecific attraction with this cue type (Figure 3d).

### 3.3 Question 3: What are the fitness benefits of conspecific attraction for habitat selection?

**FIGURE 4:**
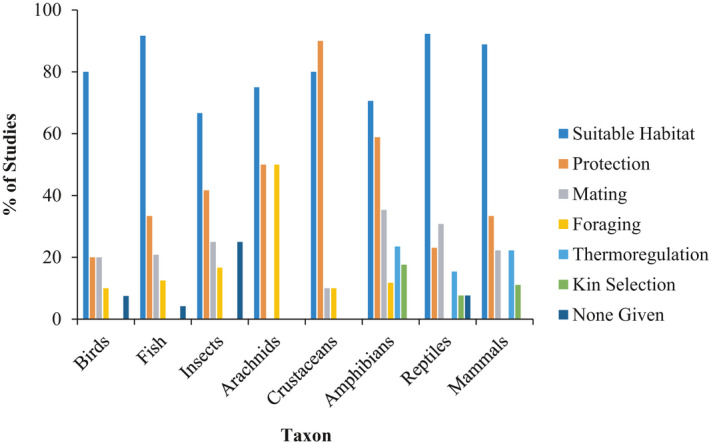
Percentage of studies citing a given ultimate explanation for conspecific attraction. Some studies included multiple explanations, while others gave no explanation (i.e., none given). Only studies that found evidence of conspecific attraction were included, as studies that did not find evidence of conspecific attraction often did not give an explanation for the behaviors

For seven of eight taxa, we found that location or identification of suitable habitat was the most commonly cited ultimate mechanism of conspecific attraction (Figure 4). Explanations of thermoregulatory benefits and kin selection were only given for amphibians, reptiles, and mammals. We found only 12 studies that quantified fitness benefits (birds = 8, crustaceans = 2, reptiles = 1, fish = 1). Among bird studies, some measure of reproductive success was the primary metric examined in relation to conspecific presence or density. Most studies in birds (*n* = 5) found no statistically significant relationship between conspecific cue presence and reproductive success (Albrecht‐Mallinger & Bulluck, 2016; Bayard & Elphick, 2012; Farrell et al., 2012; Fletcher, 2009; Harrison et al., 2009). Two bird studies found fledging success was greater at sites treated with conspecific cues compared to control sites (Anich & Ward, 2017; Ward & Schlossberg, 2004), whereas one found lower fledging success at conspecific cue sites compared to controls (Grendelmeier et al., 2017). In crustacean studies, porcelain crab (*Petrolisthes cintipes*) fitness was maximized at intermediate conspecific densities based on predation and growth rate (Donahue, 2006), whereas no relationship was found between Caribbean spiny lobster (*Panulirus argus*) survival and conspecific density (Childress & Herrnkind, 2001). Similarly, no relationship was found between survival and conspecific presence for common lizards (*Lactera vivipara*), although growth rates for juveniles were higher when conspecifics were absent (le Galliard et al., 2005). Finally, postsettlement survival of southern hulafish (*Trachinops caudimaculatus*) was greater with an increased number of conspecifics present (Fobert & Swearer, 2017).

## 4 DISCUSSION OF LITERATURE REVIEW

### 4.1 Proximate mechanisms of conspecific attraction

Our review found that a broad range of taxa exhibits conspecific attraction when selecting habitat. Depending on the taxa, ~80%–100% of studies documented conspecific attraction, suggesting the habitat selection strategy may be widespread. Notably, taxonomy diversity is considerably low among studies testing for conspecific attraction during habitat selection: To date, most experimental studies are on birds or fish, whereas crustaceans and arachnids are largely underrepresented (Figure 2). Although sample sizes within taxa are small, our review nonetheless summarizes general patterns of conspecific attraction found to date in the animal kingdom.

Conspecific attraction was most often documented in response to cue types that matched the taxa‐specific communication systems (Bradbury & Vehrencamp, 2011). For example, birds primarily communicate with acoustic cues (Kroodsma & Miller, 1996), which was the conspecific cue type that most commonly elicited conspecific attraction in this taxon (Figure 3a). Acoustic cues also elicited responses in mammals (Figure 3a). Bats comprised most mammalian studies documenting conspecific attraction, which, like birds, have sensory biases toward acoustic communication (reviewed in Gillam and Fenton 2016). Likewise, chemical cues most often elicited conspecific attraction in taxa from aquatic environments (specifically fish and crustaceans), both of which primarily use chemosensory communication systems (Sorenson & Baker, 2015). Similarly, reptiles use chemical cues to locate mates and prey (Mason & Parker, 2010), which may explain why conspecific attraction in this taxon almost exclusively occurs in response to chemical cues (Figure 3b). Visual cues have a much smaller detection radius than acoustic and chemical cues, which could be why some taxa (specifically birds, fishes, and amphibians) respond more to acoustic/chemical cues than to visual cues (Figure 3a–c). To date, almost all research on conspecific attraction focuses on taxa‐specific communication systems; fewer studies used indirect cues of conspecific presence in their experiments, such as webs (e.g., Schuck‐Paim & Alonso, 2001) or food larders (e.g., Hromada et al., 2008). The exception is for arachnids, but species samples sizes were small (*n* = 5; Figure 2) and only three studies used this cue type. More research is needed to explore how widespread conspecific attraction is in response to these indirect cues not used for communication.

We found that conspecific attraction was evident in taxa with several migratory species (such as birds and fish), or with species that exhibit breeding dispersal (such as birds, fish, and anurans). Several migratory species must quickly locate habitat upon arrival and reproduce as soon as possible, which conspecific cues could greatly assist with (Buxton et al., 2015; Danchin et al., 2004). For example, in anurans (Gatz, 1981; Wells, 2007) and birds (Amrhein et al., 2007), earlier‐arriving males are more likely to encounter and/or attract females than later‐arriving males. Similarly, spawning fish may reduce the search costs associated with long‐distance migration to spawning habitat by orienting toward conspecific cues (Bett & Hinch, 2015). Considering nonbreeding dispersal, our review found many examples of fishes (e.g., Galbraith et al., 2017; Lecchini et al., 2005; Schmucker et al., 2016; Sweatman, 1985), snakes (e.g., Burger et al., 1991; Hileman et al., 2015), and bats (Ruczyński et al., 2009; Schöner et al., 2010) that will use conspecific cues to locate nonbreeding sites, such as roosts, hibernacula, or other nonbreeding habitats. It is thus possible that dispersal, an important life history trait, shapes the likelihood of a taxon exhibiting conspecific attraction for habitat selection. Future experiments could simultaneously test multiple species from two or more taxa for conspecific attraction, including species within taxa that have different dispersal behaviors (e.g., migrants versus resident, and breeding versus nonbreeding). Including several species per taxa would be necessary to allow for phylogenetic control during comparisons, both within and between taxa.

Species‐specific mobility, which undoubtedly influences dispersal as a life history trait, could also contribute to the patterns seen in conspecific attraction across taxa. For conspecific attraction to occur, individuals must collect information on where conspecifics are present, which implicitly requires movement to sample cues. Our results reflect this potential biological necessity: Conspecific attraction is prevalent in birds and fish (Figure 2), two highly mobile groups of animals. Birds can travel long distances in short amounts of time (e.g., migration: Newton, 2007) and can disperse 1–2 km between breeding locations with relatively little effort (e.g., Betts et al., 2006). Fish are also highly mobile, with studies indicating that larvae can travel many kilometers when prospecting for breeding habitat (see Lecchini et al., 2007). Paired with a sensory bias toward potentially far‐reaching acoustic and chemical cues (discussed above), the cost to sampling conspecific cues is likely low for birds and fish. Similarly, conspecific attraction studies on mammals are heavily biased toward bats (*n* = 7 of 11 studies), which are also highly mobile and able to disperse long distances (Krauel & McCracken, 2013). A useful future direction would be to determine the perceptual ranges of species known to exhibit conspecific attraction for habitat selection, as well as how far the conspecific cue used propagates from its source in the environment. Doing so would help elucidate how accessibility of social information influences the prevalence of conspecific attraction among species that utilize it for habitat selection.

It is notable that demographic traits such as sex and age structure could influence conspecific attraction prevalence within and across taxa. However, our review revealed that few studies explicitly examined conspecific attraction by multiple age classes (*n* = 20) or by both sexes (*n* = 23) within the same study. Given that dispersal and habitat selection pressures can differ among sex and age (reviewed in Dobson, 2013), it would stand to reason that using conspecific cues for habitat selection would similarly differ. We might expect, for example, that juveniles may be more receptive to conspecific cue use because they lack experience to draw personal information from about a given habitat's suitability (e.g., Nocera et al., 2006; Ward & Schlossberg, 2004). Anecdotally, we noticed several biases in the literature prevent us from evaluating this hypothesis. Fish studies primarily tested early life stages, whereas insects primarily tested adults. Likewise, all arachnid studies were conducted on females and bird studies primarily examined male responses. Drawing on the heterospecific attraction literature, we see that social information use differs by sex in pied flycatchers (*Ficedula hypoleuca*), where females, which arrive later, were more likely to settle and pair with males in plots where blue tit (*Cyanistes caeruleus*) breeding phenology was experimentally advanced (Samplonius & Both, 2017). Future efforts should test for sex‐specific and age‐specific conspecific attraction more rigorously by: (a) designing experiments that can simultaneously monitor responses from each demographic group (male versus female, juvenile versus adult) and (b) framing predictions of which demographic group to expect conspecific attraction based on taxa‐specific life history characteristics (sensu Dobson, 2013).

Across taxa, more research is needed on how finer‐scale information embedded within conspecific cues might influence habitat selection. For example, Aragón et al. (2006) experimentally showed that the social environment of adult common lizards influenced the site selection of juveniles, indicating that individuals can assess the amount of intraspecific competition from conspecific chemical cues. Chemical cues may also contain information on conspecific body size, as Scott et al. (2013) demonstrated with small‐eyed snakes (*Cryptophis nigrescens*). In some songbirds, males sing two distinct song categories during the breeding season, with first category song sung by unpaired males and second category song sung by paired males (Spector, 1992). Kelly and Ward (2017) found that yellow warblers (*Setophaga petechia*) were more abundant at sites where second category song was experimentally broadcast, possibly because successfully paired males provide better information on habitat quality. We expect that future conspecific attraction research will advance toward understanding how information contained within a cue, and how the number of cues given influences the magnitude of attraction. In this vein, Chang et al. (2018) examined how chorus density and stimulus male call quality influenced conspecific attraction of a polyandrous choral treefrog (*Rhacophorus prasinatus*).

Future research should also address how multiple cue types exposed at once influence conspecific attraction, rather than one conspecific cue type at a time. Many organisms use multimodal signaling for communication to make fitness‐related decisions (reviewed in Hebets et al., 2016; Higham & Hebets, 2013), notably in arachnids (Herberstein et al., 2014; Uetz & Roberts, 2002), amphibians (Starnberger et al., 2014), and some birds, reptiles, and fishes (reviewed in Bro‐Jørgensen, 2010; Deodhar & Isvaran, 2018 and references therein). It is thus plausible that organisms also integrate information from several types of conspecific cues to select habitat, not just one in isolation. Surprisingly, few studies consider this possibility with their experimental paradigms, and almost all studies are on birds where both visual cues (i.e., decoys) and acoustic cues (i.e., conspecific song) are presented. This approach would be more appropriate for species that require multimodal signaling to recognize conspecifics, for example, in some insects (South et al., 2008), fishes (Hankison & Morris, 2003), and bird species (Uy et al., 2009). For species such as these, conspecific attraction may only occur if various biologically salient cues are presented in the habitat at once.

### 4.2 Fitness benefits of conspecific attraction

We found the ultimate mechanisms driving conspecific attraction, as suggested by authors for using conspecific cues, were largely the same across taxa. In practice, however, studies rarely measure the suggested fitness benefits of conspecific attraction. Indeed, of the 12 studies that attempted to measure some fitness benefit, only four found enhanced fitness in relation to conspecific presence or density. Even fewer studies discussed the costs of settling with or near conspecifics, which can include increased competition for resources (Grand & Dill, 1999), increased parasite transmission (Brown & Brown, 2004), and higher likelihood of being detected by predators (McGuire et al., 2002). This paucity of studies could be due to positive‐results bias in publication, where studies unable to find fitness benefits to conspecific attraction are less likely to get published. Lack of publications on negative results limits our ability to understand or predict directional selection for the use of conspecifics when selecting a habitat. It is thus at present difficult to tell whether (a) there are indeed fitness benefits associated with conspecific attraction that make the behavior an adaptive habitat selection strategy, or (b) conspecific attraction is not adaptive at all and is simply an exaptive byproduct of other behaviors requiring interactions with conspecifics (e.g., mating and territory defense), in line with Gould and Lewontin's famous critique of the adaptationist approach in behavioral ecology (Gould & Lewontin, 1979).

Fitness trade‐offs framed around density dependence have been discussed extensively as what has “shaped” conspecific attraction (Doligez et al., 2003; Fletcher, 2006; Szymkowiak, 2013). Given that a likely cost of settling near conspecifics is the price of having to compete with conspecific neighbors, one might expect that prospecting cue users would also assess the number of conspecifics present as part of the decision‐making process. If cue users do assess density using conspecific cues, do they prefer to settle where cues indicate high‐ or low‐density locations? Moreover, the cost of competition could preclude some individuals from using conspecific cues in high‐density locations, such as less competitive juveniles (sensu Szymkowiak et al., 2016). Surprisingly, few studies take conspecific cues revealing density into account (Kelly et al., 2018, and references therein), as most studies provide a cue that simulates one individual present at the treatment site. Fewer still compare the costs/benefits accrued by conspecific cue users to nonusers (Grendelmeier et al., 2017), making it difficult to evaluate whether conspecific attraction is an adaptive habitat selection strategy actually shaped by density dependence.

Whatever the fitness payoffs to conspecific attraction are, they are likely the sum of several information sources and not just conspecific presence. Indeed, individuals collect information from many different sources (Danchin et al., 2004; Seppänen et al., 2007; Wagner & Danchin, 2010), and in some cases, decision‐making based on information sources other than conspecifics leads to fitness higher payoffs (e.g., Doligez et al., 2003). Additionally, interactions with heterospecifics can also constrain habitat selection using conspecific cues (Fletcher, 2008; Parejo et al., 2018), which is largely ignored in the conspecific attraction literature (but see DeJong et al., 2015; Parejo & Avilés, 2016; Szymkowiak et al., 2017). Thus, rather than isolating conspecific cues, future studies should consider how individuals combine information from several sources for habitat selection (such as environmental, conspecific, and heterospecific cues), and under what scenarios conspecific cues are more useful for habitat selection than other information sources (e.g., high versus. low conspecific density, or low levels of heterospecific competition). This approach would not only help identify the selective pressures allowing for conspecific attraction to occur in a population, but could also elucidate community‐level consequences of conspecific attraction such as assembly and competitive exclusion (sensu Goodale et al., 2010).

## 5 CONCLUSIONS

The phenomenon of conspecific attraction has been a topic of interest in the scientific literature for decades, if not longer (e.g., Denton, 1889; Liley, 1982; Solomon, 1977; Stamps, 1988). In more recent decades, certain taxa, such as birds, have received considerable attention in regard to conspecific attraction (reviewed in Ahlering et al., 2010), while others have been relatively understudied. Given the > 150 studies we found on conspecific attraction, the preponderance of evidence suggests this phenomenon occurs widely across taxa for habitat selection. However, many understudied factors potentially influence conspecific attraction and it remains unclear what the fitness‐related benefits are that may ultimately drive conspecific attraction. Future studies overlaying important factors that likely shape fitness payoffs, such as life history traits (species‐specific dispersal capabilities and breeding schedules) and demographic characteristics (age and sex), will help in understanding why conspecific attraction occurs, both within and between taxa.

## CONFLICT OF INTERESTS

The authors have no competing interests regarding this review.

## AUTHOR CONTRIBUTION

**Valerie L. Buxton:** Conceptualization (equal); Data curation (equal); Formal analysis (equal); Methodology (equal); Writing‐original draft (equal). **Janice K. Enos:** Conceptualization (equal); Data curation (equal); Formal analysis (equal); Methodology (equal); Writing‐original draft (equal). **Jinelle H. Sperry:** Conceptualization (equal); Funding acquisition (lead); Project administration (equal); Resources (equal); Supervision (equal); Writing‐review & editing (equal). **Michael P. Ward:** Conceptualization (equal); Funding acquisition (supporting); Methodology (equal); Project administration (equal); Resources (equal); Supervision (equal); Writing‐review & editing (equal).

## Supporting information

Appendix S1

## Data Availability

A list of all publications included in this review is provided in Appendix S1. Data that were collated from these publications and included in the review are available at: https://doi.org/10.13012/B2IDB‐8637411_V1 (Buxton et al., 2020).
