# Supplementary material for: A review of conspecific attraction for habitat selection across taxa
Source: Ecol Evol. 2020 Nov 10;10(23):12690–9. doi: 10.1002/ece3.6922 (PMC7713925; doi:10.1002/ece3.6922)
Supplement: Supplementary file 1 — Appendix S1 [file ECE3-10-12690-s001.docx]

**Birds**

1. Ahlering MA, Johnson DH, Faaborg J (2006) Conspecific attraction in a grassland bird, the Baird’s sparrow. J Field Ornithol 77:365–371. <https://doi.org/10.1111/j.1557-9263.2006.00066.x>
2. Alatalo RV, Lindberg A, Björklund M (1892) Can the song of male birds attract other males? An experiment with the pied flycatcher *Ficedula hypoleuca*. Bird Behaviour 4:42-45.
3. Albrecht-Mallinger DJ, Bulluck LP (2016) Limited evidence for conspecific attraction in a low-density population of a declining songbird, the golden-winged warbler (*Vermivora chrysoptera*). Condor 118:451–462. <https://doi.org/10.1650/CONDOR-15-217.1>
4. Alessi MG, Benson TJ, Ward MP (2010) Nocturnal social cues attract migrating yellow-breasted chats. Wilson J Ornithol 122:780–783. <https://doi.org/10.1676/10-040.1>
5. Andrews JE, Brawn JD, Ward MP (2015) When to use social cues: Conspecific attraction at newly created grasslands. Condor 117:297–305. <https://doi.org/10.1650/CONDOR-14-172.1>
6. Anich NM, Ward MP (2017) Using audio playback to expand the geographic breeding range of an endangered species. Divers Distrib 23:1499–1508. <https://doi.org/10.1111/ddi.12635>
7. Arnold JM, Nisbet ICT, Veit R (2011) Assessing aural and visual cueing as tools for seabird management. J Wildl Manage 75:495–500. <https://doi.org/10.1002/jwmg.76>
8. Bayard TS, Elphick CS (2012) Testing for conspecific attraction in an obligate saltmarsh bird: Can behavior be used to aid marsh restoration? Wetlands 32:521–529. <https://doi.org/10.1007/s13157-012-0287-7>
9. Betts MG, Hadley AS, Rodenhouse N, Nocera JJ (2008) Social information trumps vegetation structure in breeding-site selection by a migrant songbird. Proc Biol Sci 275:2257–2263. <https://doi.org/10.1098/rspb.2008.0217>
10. Burger J (1988) Social attraction in nesting least terns : Effects of numbers, spacing, and pair bonds. Condor 90:575–582
11. Buxton RT, Jones CJ, Moller H, Lyver POB (2015) One method does not suit all: Variable settlement responses of three procellariid species to vocalisation playbacks. Emu 115:126–136. <https://doi.org/10.1071/MU14051>
12. Buxton RT, Jones IL (2012) An experimental study of social attraction in two species of storm-petrel by acoustic and olfactory cues. Condor 114:733–743. <https://doi.org/10.1525/cond.2012.110091>
13. Cornell K, Donovan T (2010) Scale-dependent mechanisms of habitat selection for a migratory passerine: An experimental approach. Auk 127:899–908
14. Farrell SL, Morrison ML, Campomizzi AJ, Wilkins RN (2012) Conspecific cues and breeding habitat selection in an endangered woodland warbler. J Anim Ecol 81:1056–1064. <https://doi.org/10.1111/j.1365-2656.2012.01995.x>
15. Fletcher RJ (2009) Does attraction to conspecifics explain the patch-size effect? An experimental test. Oikos 118:1139–1147. <https://doi.org/10.1111/j.1600-0706.2009.17342.x>
16. Fletcher RJ (2007) Species interactions and population density mediate the use of social cues for habitat selection. J Anim Ecol 76:598–606. <https://doi.org/10.1111/j.1365-2656.2007.01230.x>
17. Grendelmeier A, Arlettaz R, Olano-Marin J, Pasinelli G (2017) Experimentally provided conspecific cues boost bird territory density but not breeding performance. Behav Ecol 28:174–185. <https://doi.org/10.1093/beheco/arw144>
18. Hahn BA, Silverman ED (2007) Managing breeding forest songbirds with conspecific song playbacks. Anim Conserv 10:436–441. <https://doi.org/10.1111/j.1469-1795.2007.00128.x>
19. Hahn BA, Silverman ED (2006) Social cues facilitate habitat selection: American redstarts establish breeding territories in response to song. Biol Lett 2:337–340. <https://doi.org/10.1098/rsbl.2006.0472>
20. Harrison ML, Green DJ, Krannitz PG (2009) Conspecifics influence the settlement decisions of male Brewer’s sparrows at the northern edge of their range. Condor 111:722–729. <https://doi.org/10.1525/cond.2009.090126>
21. Jaakkonen T, Kari A, Forsman JT (2013) Flycatchers copy conspecifics in nest-site selection but neither personal experience nor frequency of tutors have an effect. PLoS One 8:e60395. <https://doi.org/10.1371/journal.pone.0060395>
22. Jaakkonen T, Kivela SM, Meier CM, Forsman JT (2015) The use and relative importance of intraspecific and interspecific social information in a bird community. Behav Ecol 26:55–64. <https://doi.org/10.1093/beheco/aru144>
23. Jeffries DS, Brunton DH (2001) Attracting endangered species to “safe” habitats: Responses of fairy terns to decoys. Anim Conserv 4:301–305. <https://doi.org/10.1017/S1367943001001354>
24. Kelly JK, Ward MP (2017) Do songbirds attend to song categories when selecting breeding habitat? A case study with a wood warbler. Behaviour 154:1123–1144. <https://doi.org/10.1163/1568539X-00003461>
25. Kotliar N, Burger J (1984) The use of decoys to attract least terns (*Sterna antillarum*) to abandoned colony sites in New Jersey. Colon Waterbirds 7:134–138. <https://doi.org/10.2307/1521092>
26. Kress SW (1983) The use of decoys, sound recordings, and gull control for a tern colony in Maine. Colonial Waterbirds 6:185–196. <https://doi.org/10.2307/1520987\>
27. Major HL, Jones IL (2011) An experimental study of the use of social information by prospecting nocturnal burrow-nesting seabirds. Condor 113:572–580. <https://doi.org/10.1525/cond.2011.100088>
28. Mariette MM, Griffith SC (2012) Conspecific attraction and nest site selection in a nomadic species, the zebra finch. Oikos 121:823–834. <https://doi.org/10.1111/j.1600-0706.2011.20014.x>
29. Mills AM, Rising JD, Jackson DA (2006) Conspecific attraction during establishment of least flycatcher cluster. J Field Ornithol 77:34–38
30. Nocera JJ, Forbes GJ, Giraldeau L-A (2006) Inadvertent social information in breeding site selection of natal dispersing birds. Proc Biol Sci 273:349–355. <https://doi.org/10.1098/rspb.2005.3318>
31. Parker MW, Kress SW, Golightly RT, Carter HR, Parsons EB, Schubel SE, Boyce JA, McChesney GJ, Wisely SM (2007) Assessment of social attraction techniques used to restore a common murre colony in central California. Waterbirds 30: 17−28.8. [https://doi.org/10.1675/1524-4695(2007)030[0017:AOSATU]2.0.CO;2](https://doi.org/10.1675/1524-4695(2007)030%5b0017:AOSATU%5d2.0.CO;2)
32. Podolsky R (1990) Effectiveness of social stimuli in attracting Laysan albatross to new potential nesting sites. Auk 107:119–124. <https://doi.org/10.2307/4087808>
33. Podolsky R, Kress SW (1989) Factors affecting colony formation in Leach’s storm-petrel. Auk 106: 332−336.
34. Podolsky R, Kress SW (1992) Attraction of the endangered dark-rumped petrel to recorded vocalizations in the Galapagos islands. Condor 94: 448−453
35. Pöysä H, Elmberg J, Sjoberg K, Nummi P (1998) Habitat selection rules in breeding mallards (*Anas platyrhynchos*): a test of two competing hypotheses. Oecologia 114:283–287. <https://doi.org/10.1007/s004420050447>
36. Quilodrán CS, Estades CF, Vásquez RA (2014) Conspecific effect on habitat selection of a territorial cavity-nesting bird. Wil J Ornithol 126:534–543
37. Rushing CS, Dudash MR, Marra PP (2015) Habitat features and long-distance dispersal modify the use of social information by a long-distance migratory bird. J Anim Ecol 84:1469–1479. <https://doi.org/10.1111/1365-2656.12395>
38. Samplonius JM, Kromhout Van Der Meer IM, Both C (2017) Nest site preference depends on the relative density of conspecifics and heterospecifics in wild birds. Front Zool 14. <https://doi.org/10.1186/s12983-017-0246-5>
39. Szymkowiak J, Thomson RL, Kuczynski L (2016) Wood warblers copy settlement decisions of poor quality conspecifics: support for the tradeoff between the benefit of social information use and competition avoidance. Oikos 125:1561–1569. <https://doi.org/10.1111/oik.03052>
40. Szymkowiak J, Thomson RL, Kuczynski L (2017) Interspecific social information use in habitat selection decisions among migrant songbirds. Behavioral Ecology 28:767–775.
41. Virzi T, Boulton RL, Davis MJ, et al (2012) Effectiveness of artificial song playback on influencing the settlement decisions of an endangered resident grassland passerine. Condor 114:846–855. <https://doi.org/10.1525/cond.2012.100197>
42. Vogel J, Koford R, Otis D (2011) Assessing the role of conspecific attraction in habitat restoration for Henslow’s sparrows in Iowa. Prairie Nat 43:23–28
43. Ward MP, Schlossberg S (2004) Conspecific attraction and the conservation of territorial songbirds. Conserv Biol 18: 519−525
44. Ward MP, Benson TJ, Semel B, Herkert JR (2010) The use of social cues in habitat selection by wetland birds. Condor 112:245–251. <https://doi.org/10.1525/cond.2010.090238>
45. Ward MP, Semel B, Jablonski C, et al (2011) Consequences of using conspecific attraction in avian conservation: A case study of endangered colonial waterbirds. Waterbirds 34:476–480. <https://doi.org/10.1675/063.034.0410>

**Amphibians**

1. Bee MA (2007) Selective phonotaxis by male wood frogs (*Rana sylvatica*) to the sound of a chorus. Behav Ecol Sociobiol 61:955–966. <https://doi.org/10.1007/s00265-006-0324-8>
2. Buxton VL, Ward MP, Sperry JH (2015) Use of chorus sounds for location of breeding habitat in 2 species of anuran amphibians. Behav Ecol 26:1111–1118. <https://doi.org/10.1093/beheco/arv059>
3. Chapman TL, Holcomb MP, Spivey KL, Sehr EK, Gall BG (2014) A test of local enhancement in amphibians. Ethology 120:1–7. <https://doi.org/10.1111/eth.12337>
4. Christie K, Schul J, Feng AS (2010) Phonotaxis to male’s calls embedded within a chorus by female gray treefrogs, *Hyla versicolor*. J Comp Physiol A 196:569–79. <https://doi.org/10.1007/s00359-010-0544-2>
5. Gautier P, Olgun K, Uzum N, Miaud C (2006) Gregarious behaviour in a salamander: attraction to conspecific chemical cues in burrow choice. Behav Ecol Sociobiol 59:836–841. <https://doi.org/10.1007/s00265-005-0130-8>
6. Gerhardt HC, Klump GM (1988) Phonotactic responses and selectivity of barking treefrogs (*Hyla gratiosa*) to chorus sounds. J Comp Physiol A 163:795–802. <https://doi.org/10.1007/BF00604056>
7. Gonzalo A, Cabido C, Galan P, et al (2006) Predator, but not conspecific, chemical cues influence pond selection by recently metamorphosed Iberian green frogs, *Rana perezi*. Can J Zool 1299:1295–1299. <https://doi.org/10.1139/Z06-118>
8. Graves BM, Summers CH, Olmstead KL (1993) Sensory mediation of aggregation among postmetamorphic *Bufo cognatus*. J Herpetol 27:315–319. <https://doi.org/10.2307/1565153>
9. Greene KM, Pittman SE, Dorcas ME (2016) The effects of conspecifics on burrow selection in juvenile spotted salamanders (*Ambystoma maculatum*). J Ethol 34:309–314. <https://doi.org/10.1007/s10164-016-0476-6>
10. Hamer R, Lemckert FL, Banks PB (2011) Adult frogs are sensitive to the predation risks of olfactory communication. Biol Lett 7:361–3. <https://doi.org/10.1098/rsbl.2010.1127>
11. Höbel G, Christie A (2016) Do green treefrogs use social information to orient outside the breeding season? Zool Stud 55:1–8. <https://doi.org/10.6620/ZS.2016.55-17>
12. James MS, Stockwell MP, Clulow J, Clulow S, Mahony MJ (2015) Investigating behaviour for conservation goals: Conspecific call playback can be used to alter amphibian distributions within ponds. Biol Conserv 192:287–293. <https://doi.org/10.1016/j.biocon.2015.10.001>
13. Lea J, Dyson M, Halliday T (2002) Phonotaxis to advertisement calls by midwife toads (*Alytes muletensis*) is not necessarily related to mating. Amphibia-Reptilia 23:151–159. <https://doi.org/10.1163/156853802760061796>
14. Leu ST, Whiting MJ, Mahony MJ (2013) Making friends: Social attraction in larval green and golden bell frogs, *Litoria aurea*. PLoS One 8:e56460. <https://doi.org/10.1371/journal.pone.0056460>
15. Pfennig KS, Rapa K, McNatt R (2000) Evolution of male mating behavior: male spadefoot toads preferentially associate with conspecific males. Behav Ecol Sociobiol 48:69–74. <https://doi.org/10.1007/s002650000205>
16. Pizzatto L, Stockwell M, Clulow S, et al (2016) Finding a place to live: conspecific attraction affects habitat selection in juvenile green and golden bell frogs. Acta Ethol 19:1–8. <https://doi.org/10.1007/s10211-015-0218-8>
17. Secondi J, Haerty W, Lode T (2005) Female attraction to conspecific chemical cues in the palmate newt *Triturus helveticus.* Ethology 111:726–735. <https://doi.org/10.1111/j.1439-0310.2005.01096.x>
18. Swanson EM, Tekmen SM, Bee MA (2007) Do female frogs exploit inadvertent social information to locate breeding aggregations? Can J Zool 85:921–932. <https://doi.org/10.1139/Z07-074>
19. Verrell P, Davis K (2003) Do non-breeding, adult long-toed salamanders respond to conspecifics as friends or as foes? Herpetologica 59:1−7
20. Vignoli L, Silici R, Bissattini AM, Bologna MA (2012) Aspects of olfactory mediated orientation and communication in *Salamandrina perspicillata* (Amphibia Caudata): An experimental approach. Ethol Ecol Evol 24:165–173. <https://doi.org/10.1080/03949370.2011.591437>

**Insects**

1. Byers CJ, Eason PK (2009) Conspecifics and their posture influence site choice and oviposition in the damselfly *Argia moesta*. Ethology 115:721–730. <https://doi.org/10.1111/j.1439-0310.2009.01658.x>
2. Dukas R, Prokopy RJ, Papaj DR, Duan JJ (2001) Egg laying behavior of mediterranean fruit flies (Diptera: Tephritidae): is social facilitation important? Florida Entomol 84:665–671
3. Grether GF, Switzer PV. (2000) Mechanisms for the formation and maintenance of traditional night roost aggregations in a territorial damselfly. Anim Behav 60:569–579. <https://doi.org/10.1006/anbe.2000.1511>
4. Grevstad FS, Herzig AL (1997) Quantifying the effects of distance and conspeciﬁcs on colonization: experiments and models using the loosestrife leaf beetle, *Galerucella calmariensis*. Oecologia 110:60–68. <https://doi.org/10.1007/s004420050133>
5. Martens A (1989) Aggregation of tandems in *Coenagrion pulchellum* during oviposition (Odonata: Coenagrionidae). Zool Anz 223:124–128
6. Martens, A. and Rehfeldt, G. 1989. Female aggregation in *Platychypha caligata* (Odonata: Chlorocyphidae): a tactic to evade male interference during oviposition. − Anim. Behav. 38: 369−374.
7. Martens, A. 1993. Influence of conspecifics and plant structures on oviposition site selection in *Pyrrhosoma nymphula* (sulzer) (Zygoptera: Coenagrionidae). − Odonatologica 22: 487−494.
8. Martens, A. 1994. Field experiments on aggregation behaviour and oviposition in *Coenagrion puella* (L.) (Zygoptera: Coenagrionidae). − Adv. Odonatol. 6: 49−58.
9. Miller CW, Fletcher RJ, Gillespie SR (2013) Conspecific and heterospecific cues override resource quality to influence offspring production. PLoS One 8:1–7. <https://doi.org/10.1371/journal.pone.0070268>
10. Muller KL (1998) The role of conspecifics in habitat settlement in a territorial grasshopper. Anim Behav 56:479–485. <https://doi.org/10.1006/anbe.1998.0806>
11. Norris (1963) Laboratory experiments on gregarious behavior in ovipositing females of the desert locust (*Schistocerca gregaria* (Forsk.)). Ent Exp & Appl 6: 279−303.
12. Norris MJ (1970) Aggregation response in ovipositing females of the desert locust, with special reference to the chemical factor. J Insect Physiol 16:1493–1515. <https://doi.org/10.1016/0022-1910(70)90249-0>
13. Prokopy RJ, Miller NW, Duan JJ, Vargas RI (2000) Local enhancement of arrivals of *Ceratitis capitata* females on fruit mimics. Entomol Exp Appl 97:211–217. <https://doi.org/10.1023/A:1004064005423>
14. Prokopy RJ, Romig MC, Drew RAI (1999) Facilitation in ovipositional behavior of *Bactrocera tryoni flies*. J Insect Behav 12:815–832. <https://doi.org/10.1023/a:1020909227680>
15. Waage JK (1987) Choice and utilization of oviposition sites by female *Calopteryx maculata* (Odonata: Calopterygidae). Behav Ecol Sociobiol 20:439–446. <https://doi.org/10.1007/BF00302987>

**Reptiles**

1. Aragón P, López P, Martín J (2001) Effects of conspecific chemical cues on settlement and retreat-site selection of male lizards *Lacerta monticola*. J Herpetol 35:681–684.
2. Aragón P, Massot M, Gasparini J, Clobert J (2006) Socially acquired information from chemical cues in the common lizard, *Lacerta vivipara*. Anim Behav 72:965–974. <https://doi.org/10.1016/j.anbehav.2005.11.023>
3. Brown WS, MacLean FM (1983) Conspecific scent-trailing by newborn timber rattlesnakes, *Crotalus horridus*. Herpetologica 39:430–436
4. Bulova SJ (1997) Conspecific chemical cues influence burrow choice by desert tortoises (*Gopherus agassizii*). Copeia 1997:802–810. <https://doi.org/10.2307/1447297>
5. Burger J (1989) Following of conspecific and avoidance of predator chemical cues by pine snakes (*Pituophis melanoleucus*). J Chem Ecol 15:799–806. <https://doi.org/10.1007/BF01015178>
6. Burger J, Boarman W, Kurzava L, Gochfiled M (1991) Effect of experience with pine (*Pituophis melanoleucus*) and king (*Lampropelitis getulus*) snake odors on y-maze behavior of pine snake hatchlings. J Chem Ecol 17:79–87
7. Costanzo JP (1989) Conspecific scent trailing by garter snakes (*Thamnophis sirtalis*) during autumn: Further evidence for use of pheromones in den location. J Chem Ecol 15:2531–2538. <https://doi.org/10.1007/BF01014729>
8. Hileman ET, Bradke DR, Delaney DM, King RB (2015) Protection by association: implications of scent trailing in neonate eastern massasaugas. Herpetol Conserv Biol 10:654–660
9. Lawson PA (1994) Orientation abilities and mechanisms in nonmigratory populations of garter snakes (*Thamnophis sirtalis* and *T. ordinoides*). Copeia 1994:263–274. <https://doi.org/10.1038/1391014a0>
10. Le Galliard JF, Ferrière R, Clobert J (2005) Effect of patch occupancy on immigration in the common lizard. J Anim Ecol 74:241–249. <https://doi.org/10.1111/j.1365-2656.2004.00912.x>
11. LeMaster MP, Moore IT, Mason RT (2001) Conspecific trailing behaviour of red-sided garter snakes, Thamnophis sirtalis parietalis, in the natural environment. Anim Behav 61:827–833. <https://doi.org/10.1006/anbe.2000.1658>
12. Muñoz A (2004) Chemo-orientation using conspecific chemical cues in the stripe-necked terrapin (*Mauremys leprosa*). J Chem Ecol 30:519–530. <https://doi.org/10.1023/B:JOEC.0000018626.55609.31>
13. Porter RH, Czaplicki JA (1974) Responses of water snakes (*Natrix r. rhombifera*) and garter snakes (*Thamnophis sirtalis*) to chemical cues. 2:129–132
14. Quinn VS, Graves BM (1998) Home pond discrimination using chemical cues in *Chrysemys picta*. J Herp 32: 457−461.
15. Scott ML, Whiting MJ, Webb JK, Shine R (2013) Chemosensory discrimination of social cues mediates space use in snakes, *Cryptophis nigrescens* (Elapidae). Anim Behav 85:1493–1500. <https://doi.org/10.1016/j.anbehav.2013.04.003>
16. Smith GR, Iverson JB (1993) Reactions to odor trails in bullsnakes. J Herp 27: 333−335
17. Stamps JA (1987) Conspecifics as cues to territory quality: a preference of juvenile lizards (*Anolis aeneus*) for previously used territories. Am Nat 129:629–642
18. Stamps JA (1988) Conspecific attraction and aggregation in territorial species. Am Nat 131:329-347.

**Mammals**

1. Arnold BD, Wilkinson GS (2011) Individual specific contact calls of pallid bats (*Antrozous pallidus*) attract conspecifics at roosting sites. Behav Ecol Sociobiol 65:1581–1593. <https://doi.org/10.1007/s00265-011-1168-4>
2. Cassaing J, Le Proux De La Riviere B, De Donno F, Marlinez-Garcia E, Thomas C (2013) Interactions between 2 Mediterranean rodent species: Habitat overlap and use of heterospecific cues. Ecoscience 20:137–147. <https://doi.org/10.2980/20-2-3560>
3. Chaverri G, Gillam EH, Vonhof MJ (2010) Social calls used by a leaf-roosting bat to signal location. Biol Lett 6:441–444. <https://doi.org/10.1098/rsbl.2009.0964>
4. Fisher DO, Lambin X, Yletyinen SM (2008) Experimental translocation of juvenile water voles in a Scottish lowland metapopulation. Popul Ecol 51:289–295. <https://doi.org/10.1007/s10144-008-0122-4>
5. Furmankiewicz J, Ruczyński I, Urban R, Jones G (2011) Social calls provide tree-dwelling bats with information about the location of conspecifics at roosts. Ethology 117:480–489. <https://doi.org/10.1111/j.1439-0310.2011.01897.x>
6. Glorvigen P, Bjørnstad ON, Andreassen HP, Ims RA (2012) Settlement in empty versus occupied habitats: an experimental study on bank voles. Popul Ecol 54:55–63. <https://doi.org/10.1007/s10144-011-0295-0>
7. Kerth G, Reckardt K (2003) Information transfer about roosts in female Bechstein’s bats: an experimental field study. Proc R Soc B Biol Sci 270:511–515. <https://doi.org/10.1098/rspb.2002.2267>
8. McGuire B, Oli MK, Getz LL (2009) Effects of conspecific and heterospecific residents on patterns of immigration in two species of voles. Acta Theriol 54:321–332. <https://doi.org/10.4098/j.at.0001-7051.107.2008>
9. Ruczyński I, Kalko EKV, Siemers BM (2009) Calls in the forest: A comparative approach to how bats find tree cavities. Ethology 115:167–177. <https://doi.org/10.1111/j.1439-0310.2008.01599.x>
10. Ruczynski I, Kalko EK V., Siemers BM (2007) The sensory basis of roost finding in a forest bat, *Nyctalus noctula*. J Exp Biol 210:3607–3615. <https://doi.org/10.1242/jeb.009837>
11. Schöner CR, Schöner MG, Kerth G (2010) Similar is not the same: Social calls of conspecifics are more effective in attracting wild bats to day roosts than those of other bat species. Behav Ecol Sociobiol 64:2053–2063. <https://doi.org/10.1007/s00265-010-1019-8>

**Fishes**

1. Baker CF, Hicks BJ (2003) Attraction of migratory inanga (*Galaxias maculatus*) and koaro (*Galaxias brevipinnis*) juveniles to adult galaxiid odours. New Zeal J Mar Freshw Res 37:291–299. <https://doi.org/10.1080/00288330.2003.9517167>
2. Baker CF, Montgomery JC (2001) Species-specific attraction of migratory banded kokopu juveniles to adult pheromones. J Fish Biol 58:1221–1229. <https://doi.org/10.1006/jfbi.2000.1528>
3. Bett NN, Hinch SG (2015) Attraction of migrating adult sockeye salmon to conspecifics in the absence of natal chemical cues. Behav Ecol 26:1180–1187. <https://doi.org/10.1093/beheco/arv062>
4. Black GA, Dempson JB (1986) A test of the hypothesis of pheromone attraction in salmonid migration. Environ Biol Fishes 15:229–235. <https://doi.org/10.1007/BF00002996>
5. Booth DJ (1992) Larval settlement patterns and preferences by domino damselfish *Dascyllus albisella*. J Exp Mar Bio Ecol 155:85–104. <https://doi.org/10.1016/0022-0981(92)90029-A>
6. Coppock AG, Gardiner NM, Jones GP (2013) Olfactory discrimination in juvenile coral reef fishes: Response to conspecifics and corals. J Exp Mar Bio Ecol 443:21–26. <https://doi.org/10.1016/j.jembe.2013.02.026>
7. Coppock AG, Gardiner NM, Jones GP (2016) Sniffing out the competition? Juvenile coral reef damselfishes use chemical cues to distinguish the presence of conspecific and heterospecific aggregations. Behav Processes 125:43–50. <https://doi.org/10.1016/j.beproc.2016.02.001>
8. Elvidge CK, Cooke ELL, Cunjak RA, Cooke SJ (2017) Social cues may advertise habitat quality to refuge-seeking conspecifics. 5:1–5. <https://doi.org/10.1139/cjz-2016-0144>
9. Fobert EK, Swearer SE (2017) The nose knows: linking sensory cue use, settlement decisions, and post-settlement survival in a temperate reef fish. Oecologia 183:1041–1051. <https://doi.org/10.1007/s00442-017-3843-2>
10. Galbraith HS, Blakeslee CJ, Schmucker AK, Johnson NS, Hansen MJ, Li W (2017) Donor life stage influences juvenile American eel *Anguilla rostrata* attraction to conspecific chemical cues. J Fish Biol 90:384–395. <https://doi.org/10.1111/jfb.13190>
11. Gardiner NM, Jones GP (2010) Synergistic effects of habitat preference and gregarious behaviour on habitat use in coral reef cardinalfish. Coral Reefs 29:845–856. <https://doi.org/10.1007/s00338-010-0642-1>
12. Groot C, Quinn TP, Hara TJ (1986) Responses of migrating adult sockeye salmon (*Oncorhynchus nerka*) to population-specific odours. Can J Zool 64:926–932
13. Hale R, Swearer SE, Downes BJ (2009) Separating natural responses from experimental artefacts: Habitat selection by a diadromous fish species using odours from conspecifics and natural stream water. Oecologia 159:679–687. <https://doi.org/10.1007/s00442-008-1248-y>
14. Huijbers CM, Nagelkerken I, Lössbroek PAC, Schulten IE, Siegenthaler A, Holderied MW, Simpson SD (2012) A test of the senses: Fish select novel habitats by responding to multiple cues. Ecology 93:46–55. <https://doi.org/10.1890/10-2236.1>
15. Igulu MM, Nagelkerken I, Fraaije R, van Hintum R, Ligtenberg H, Mgaya YD (2011) The potential role of visual cues for microhabitat selection during the early life phase of a coral reef fish (*Lutjanus fulviflamma*). J Exp Mar Bio Ecol 401:118–125. <https://doi.org/10.1016/j.jembe.2011.01.022>
16. Igulu MM, Nagelkerken I, van der Beek M, Schippers M, van Eck R, Mgaya YD (2013) Orientation from open water to settlement habitats by coral reef fish: behavioral flexibility in the use of multiple reliable cues. Marine Ecology Progress Series 493:243–257.
17. Lecchini D, Nakamura Y (2013) Use of chemical cues by coral reef animal larvae for habitat selection. Aquat Biol 19:231–238. <https://doi.org/10.3354/ab00532>
18. Lecchini D, Osenberg CW, Shima JS, St Mary CM, Galzin R (2007) Ontogenetic changes in habitat selection during settlement in a coral reef fish: Ecological determinants and sensory mechanisms. Coral Reefs 26:423–432. <https://doi.org/10.1007/s00338-007-0212-3>
19. Lecchini D, Planes S, Galzin R (2005) Experimental assessment of sensory modalities of coral-reef fish larvae in the recognition of their settlement habitat. Behav Ecol Sociobiol 58:18–26. <https://doi.org/10.1007/s00265-004-0905-3>
20. Lecchini D, Shima J, Banaigs B, Galzin R (2005) Larval sensory abilities and mechanisms of habitat selection of a coral reef fish during settlement. Oecologia 143:326–334. <https://doi.org/10.1007/s00442-004-1805-y>
21. Levin PS (1993) Habitat structure, conspecific presence and spatial variation in the recruitment of temperate reef fish. Oecologia 94:176–185
22. Quinn TP, Brannon EL, Whitman RP (1983) Pheromones and the water source of adult coho salmon (*Oncorhynchus kisutch*) Walbaum. J Fish Biol 22:677–684
23. Quinn TP, Tolson GM (1986) Evidence of chemically mediated population recognition in coho salmon (*Oncorhynchus kisutch*). Can J Zool 64:84–87. <https://doi.org/10.1139/z86-013>
24. Schmucker AK, Johnson NS, Galbraith HS, Li W (2016) Glass-eel-stage American eels respond to conspecific odor as a function of concentration. Trans Am Fish Soc 145:712–722. <https://doi.org/10.1080/00028487.2016.1146164>
25. Sweatman HPA (1983) Influence of conspecifics on choice of settlement sites by larvae of two pomacentrid fishes (*Dascyllus aruanus* and *D. reticulatus*) on coral reefs. Mar Biol 75:225–229. <https://doi.org/10.1007/BF00406006>
26. Sweatman HPA (1985) The influence of adults of some coral reef fishes on larval recruitment. Ecological Monogr 55:469–485
27. Sweatman HPA (1988) Field evidence that settling coral reef fish larvae detect resident fishes using dissolved chemical cues. J Exp Mar Bio Ecol 124:163–174. <https://doi.org/10.1016/0022-0981(88)90170-0>

**Crustaceans**

1. Briones-Fourzán P, Ramírez-Zaldívar E, Lozano-Álvarez E (2008) Influence of conspecific and heterospecific aggregation cues and alarm odors on shelter choice by syntopic spiny lobsters. Biol Bull 215:182–190.
2. Childress MJ, Herrkind WF (2001) The guide effect influence on the gregariousness of juvenile Caribbean spiny lobsters. Anim Behav 62:465–472. <https://doi.org/10.1071/MF01047>
3. Donahue MJ (2006) Allee effects and conspecific cueing jointly lead to conspecific attraction. Oecologia 149:33–43. <https://doi.org/10.1007/s00442-006-0419-y>
4. Grove MW, Woodin SA (1996) Conspecific recognition and host choice in a pea crab, *Pinnixa chaetopterana* (Brachyura-Pinnotheridae). Biol Bull 190:359–366
5. Jensen C (1989) Gregarious settlement by megalopae of the porcelain crabs *Petrolisthes cinctipes* (Randall) and P. eriomerus Stimpson. J Exp Mar Biol Ecol 131:223–231
6. Jensen GC (1991) Competency, settling behavior, and postsettlement aggregation by porcelain crab megalopae (Anomura: Porcellanidae). J Exp Mar Biol Ecol 153:49–61
7. Nevitt G, Pentcheff ND, Lohmann KJ, Zimmer RK (2000) Den selection by the spiny lobster. Mar Ecol Prog Ser 203:225–231. <https://doi.org/10.3354/meps203225>
8. Ratchford SG, Eggleston DB (1998) Size- and scale-dependent chemical attraction contribute to an ontogenetic shift in sociality. Anim Behav 56:1027–1034. <https://doi.org/10.1006/anbe.1998.0869>
9. Zimmer-faust RK (1985) Chemical attraction causing aggregation in the spiny lobster, *Panulirus interruptus*, and its probably ecological significance. Biol Bull 169:106–118
10. Zimmer-Faust RK, Spanier E (1987) Gregariousness and sociality in spiny lobsters: implications for den habitation. J Exp Mar Bio Ecol 105:57–71. https://doi.org/10.1016/S0022-0981(87)80029-1

**Arachnids**

1. Clotuche G, Mailleux A-C, Yano S, Detrain C, Deneubourg J-L, Hance T (2013) Settlement decisions by the two-spotted spider mite *Tetranychus urticae*. C R Biol 336:93–101. <https://doi.org/10.1016/j.crvi.2013.02.006>
2. Fitzgerald MR, Ives AR (2017) Conspecific attraction drives intraspecific aggregations by *Nephila clavipes* spiders. Ethology 123:51–60. <https://doi.org/10.1111/eth.12577>
3. Hodge MA, Storfer-Isser A (1997) Conspecific and heterospecific attraction: A mechanism of web-site selection leading to aggregation formation by web-building spiders. Ethology 103:815–826. <https://doi.org/10.1111/j.1439-0310.1997.tb00123.x>
4. Penfold S, Dayananda B, Webb JK (2017) Chemical cues influence retreat-site selection by flat rock spiders. Behaviour 154:149–161. <https://doi.org/http://dx.doi.org/10.1163/1568539X-00003415>
5. Schuck-Paim C, Alonso WJ (2001) Deciding where to settle: conspecific attraction and web site selection in the orb-web spider *Nephilengys cruentata*. Anim Behav 62:1007–1012. <https://doi.org/10.1006/anbe.2001.1841>
